# Comparing program supervision with an external RADAR evaluation of quality of care in integrated community case management for childhood illnesses in Mali

**DOI:** 10.1080/16549716.2021.2006424

**Published:** 2022-09-13

**Authors:** Luay Basil, Mary Thompson, Melissa A. Marx, Emily Frost, Diwakar Mohan, Sinaly Traore, Jules Zanre, Bintou Coulibaly, Birahim Yagyemar Gueye, Thierry Nkurabagaya, Ghislain Poda, Kone Moussa, Farida El-Kalaawy, Christina Angelaksi

**Affiliations:** a Global Programs, Canadian Red Cross, Ottawa, Canada; b Department of International Health, Johns Hopkins Bloomberg School of Public Health, Johns Hopkins University, Baltimore, Maryland, USA; c Office of Science and Strategy, March of Dimes, Arlington, VA, USA; d Department of Health, Malian Red Cross, Bamako, Mali

**Keywords:** Integrated community case management, community health worker, quality of care, program supervision, RADAR

## Abstract

**Background:**

Many countries have adopted integrated community case management (iCCM) to reduce mortality among children under five years from common childhood illnesses. The 2016–2020 Malian Red Cross iCCM program trained 441 Community Health Workers (CHWs) to treat malaria, pneumonia, diarrhea, and malnutrition for children under five years of age in six districts. Implementation strength and quality of care (QoC) were assessed through the program’s supervision function, using the Malian Ministry of Health’s system.

**Objective:**

This paper compares methods and results of program supervision data and an independent evaluation to assess the effectiveness of program implementation and supervision and inform program improvement. It also presents the benefits and limitations of each method.

**Method:**

An independent QoC evaluation was conducted using tools developed by the Real Accountability: Data Analysis for Results (RADAR) project, hereafter referred to as the *RADAR evaluation*. RADAR evaluation data collected in July and August 2018 were compared with program supervision data collected mostly between May and December 2018.

**Results:**

The RADAR evaluation provided detailed findings on correct assessment, classification, and treatment per illness, medication type, and dosage. Program supervision combined the findings for all illnesses, medication type, and dosage due to limitations in the data collection process. Six indicators were comparable between both methods. Findings were similar for temperature and mid-upper arm circumference measurements but diverged between program supervision and the RADAR evaluation, respectively, on correct classification for all illnesses (87.1% vs. 65.3%), correct treatment for all illnesses (69.5% vs. 39.8%), correct respiratory rate counting (88.5% vs. 54.7%), and administering the first dose by CHW (75.4% vs. 65.0%). Findings from the RADAR evaluation guided improvements in program supervision.

**Conclusions:**

A robust program supervision system can serve as a credible method to assess QoC. However, a rigorous independent QoC evaluation provides a valuable benchmark to gauge the effectiveness of the supervisory process.

## Background

Integrated community case management (iCCM) is a strategy endorsed by WHO and UNICEF to reduce mortality among children under five years by increasing access to services for common childhood illness [1]. Over 30 countries using iCCM have trained and equipped community health workers (CHWs) to diagnose and treat children against malaria, diarrhea, and pneumonia [2]. Supervision, periodic refresher trainings, and performance quality assurance are critical components of a strong and effective iCCM program and key to maintaining and enhancing CHWs’ skills in managing childhood illness [1,3,4].

A review of 22 studies conducted in 2014 on iCCM impact and implementation considered supportive supervision as best practice, with defined elements such as record reviews, case management observations, constructive feedback, provider participation, problem-solving, and focused education influencing CHWs’ performance, motivation, and retention [5]. Other studies highlighted data collection, coaching, and on-the-spot training [3,6]. High-quality supervision by formal health workers legitimizes CHWs in the eyes of other health workers and the communities served and is an important means of integrating CHWs within the public health system [5,7].

Improving supervision quality by ensuring that it includes key elements of supportive supervision – particularly performance monitoring, constructive feedback, problem-solving, and focused education – has a greater impact than increasing frequency of supervisions that lack such elements [5]. A dose-response relationship between the number of supportive supervision visits and the consistency of iCCM skills of health extension workers has been observed [8].

Supportive supervision is a process of helping workers improve their own work performance continuously [9]. It occurs in multiple places: on the job, both formally and informally; in one-on-one meetings; in peer discussions; in meetings outside the workplace; and when health workers review their own performance against standards [10]. Supervision extends beyond formal site visits to the ongoing relationship between a healthcare provider and supervisor, with the latter acting as the facilitator, trainer, and coach [10].

In practice, however, supportive supervision is often weak and under-supported [5]. In a review of 20 iCCM programs in East and Southern Africa, lack of sufficient supportive supervision was one of the most commonly mentioned challenges, due to low availability and/or capacity of supervisors and/or no incentives for supervisors or CHWs to participate in supervisory visits [7]. Supportive supervision also requires that supervisors are trained in problem identification, problem-solving, time management, communication, monitoring, coaching, and technical and clinical updates [11].

Mali’s National Child Strategy 2007–2012 provided a strategic plan for newborn and child survival, including a full iCCM package and acknowledging for the first time the CHW’s role in delivering community case management. The strategy’s original tools and guidelines for CHWs’ training, service delivery, and supervision were revised in 2015. In 2016, the Malian Red Cross, supported by the Canadian Red Cross, scaled up its iCCM program (Improving Maternal, Newborn, and Child Health in Mali) to a second five-year phase and enhanced its supervision methods. It used the Ministry of Health’s (MoH) iCCM training curriculum and new tools, templates, and guidelines, including *Essential Care in the Community*, (the main guide for CHWs) and the *Supervision Evaluation Form* [Supplement Figure 1].

**Figure 1.**
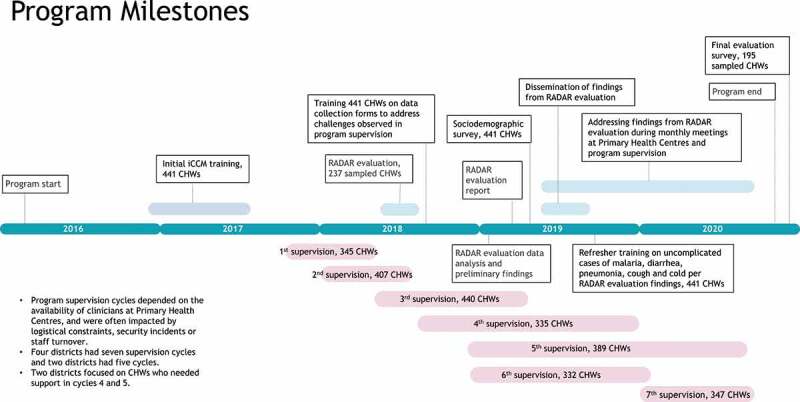
Program milestones.

The iCCM program trained 441 CHWs in rural communities in Koulikoro and Sikasso regions to provide preventive and curative care to children from 2 to 59 months of age for uncomplicated malaria, diarrhea, pneumonia, and malnutrition, as well as newborn care and family planning services. These CHWs covered 756 villages totaling around 650,000 people in six districts and were supervised by clinicians working in 130 Primary Health Centers (PHCs).

The program hired District Counsellors (doctors or nurses) to mentor CHWs and provide technical support to the PHCs in supervising the CHWs. After initial training, some PHCs kept their CHWs for one to two weeks at the facility to solidify their technical skills and strengthen the relationship between the CHWs and PHC personnel.

The Real Accountability: Data Analysis for Results (RADAR) project conducted an independent, cross-sectional evaluation of the iCCM program (the RADAR evaluation) to measure the implementation strength and QoC provided by participating CHWs [12]. The RADAR evaluation was carried out in partnership with the *Centre de Recherche, d’Études et de Documentation pour la Suivie de l’Enfant (CREDOS*) between July and August 2018. While supervision methods and the RADAR evaluation assessed both implementation strength (availability of medical equipment, records, and commodities, and their storage condition) and QoC (correct classification and treatment), this paper focuses on QoC for childhood illnesses.

This paper compares the methods and results of the Red Cross program’s supervision data and the RADAR evaluation to inform program improvement around QoC provided by CHWs. It also presents the benefits and limitations of using each method.

This paper is part of the supplement “Strengthening Effectiveness Evaluations to Improve Programs for Women and Children”. The paper in the supplement “Marx MA et al. Tools and methods to Measure the Quality of Care for Maternal, Neonatal, Child, Reproductive Health and Nutrition Programs: Guidance from use in four sub Saharan African Countries through the RADAR project” describes the Quality of Care evaluation tool used in Mali.

## Methods

We compared findings from program supervision with those from the RADAR evaluation to assess the impact of supervision on QoC, which was provided by CHWs. We hypothesized that the closer the estimates are between similar indicators from program supervision data and from a rigorous, external evaluation, the more effective the program supervision in assessing the CHWs’ QoC level and improving it; wider findings indicate that the supervision process requires adjustments.

## Data sources

The study takes a data triangulation approach using the following secondary data sources.

- Longitudinal secondary data collected during program supervision between 2017 and 2020 using the MoH’s iCCM strategy to evaluate QoC.
- Cross-sectional secondary data from the RADAR evaluation using tools developed by the RADAR project.
- A 2019 socio-demographic program survey of all CHWs that included findings on the relationship between CHWs and their supervisors.
- A survey of sampled CHWs as part of the iCCM program’s final evaluation that included findings on CHWs’ perception of program supervision.

## Program supervision

The MoH program supervision of the CHWs by the PHC should be performed monthly, using the *Supervision Evaluation Form*. However, such frequency is not practical due to the lack of adequate financing. In this paper, ‘program supervision’ is defined as the program’s routine, supportive supervision. Supervision teams included three to nine members, but always at a minimum, the chief clinician from each PHC, a representative from the Community Health Association, and the program’s District Counsellor.

The program intended to adhere to MoH supervision guidelines, but it took more than three months to complete a supervision cycle in a district depending on the resources available, logistical constraints, and security incidents. As a result, some districts had fewer supervision cycles than others. During seven supervision cycles (October 2017 to September 2020), the program conducted 2,705 supervisory visits, 440 of which were in Cycle 3 (Figure 1). Data was collected using the *Supervision Evaluation Form* (Supplement Figure 1).

CHWs in Mali are mandated to provide services in the following areas: childhood illnesses, newborn care, infant and child feeding practices, family planning, and hygiene and sanitation. Program supervision evaluated QoC provided by CHWs through records review and direct observation of case management. Records review measured five indicators on childhood illnesses, while observation measured 39 indicators of the full CHW mandate, of which 13 were on childhood illness. The MoH system uses the 39 indicators as a composite indicator, converting it to a scale of 100 points. CHWs with 80% scoring or more on the composite indicator demonstrated a satisfactory level of performance of their mandate. We identified 11 indicators from records review and observation of case management that were relevant to our objective (Supplement Table 1).

**Table 1.** Comparison between the methodologies of program supervision and the RADAR evaluation.

Program supervision	RADAR Evaluation
**Evaluators**	
130 PHC clinicians trained to supervise CHWs in their catchment areas. On average, two clinicians consistently supervised three to four CHWs.	20 trained clinicians used as the gold standard and 10 supervisors.
**Number of CHWs evaluated**	
Cycle 3 was used for the RADAR comparison, whereby 440 CHWs out of the 441 in the program were supervised. CHWs were supervised between five to seven times during the program. Two districts adopted a targeted approach focusing on lower performing CHWs in Cycle 4 (Kolokani and Sikasso) and Cycle 5 (Sikasso).See Supplement Table 2.	Of CHWs working in 441 functional iCCM sites, 300 were selected using a stratified random sample proportional to the number of CHWs in each district. From the stratified sampling scheme, the CHW distribution was per the number of CHW’s working in the district. The sample size provided an estimate of 50% prevalence with 6% precision and a type I error of 0.05, with 12% refusal to participate. Due to access and security constraints, only 237 CHWs were evaluated.
**Consent**	
Consent was obtained from the sick child’s companion in case management observations and in simulations. If the companion was under 18 years of age and was not a parent, the supervisor and the CHW asked for a related adult to give consent. In the absence of that, another sick child was sought.	Consent was obtained from the CHW and the sick child’s companion before conducting the evaluation, including from mothers between 15 and 17 years of age.
**Evaluation of quality of care**	
A clinician observed a CHW examining a sick child brought by a companion and recorded the observations in accordance with the MoH protocol (assessment of symptoms and danger signs, classification, treatment, or referral).CHW accuracy in measuring the respiratory rate was determined by the supervisor and the CHW simultaneously counting the child’s breaths using a timer and the CHW first revealing their count to the supervisor. The count by the CHW was considered correct if it was within 2 breaths of the count of the supervisor’s.Evaluated correct usage of the rapid diagnostic kit to classify malaria.CHW treatment was considered correct if both the prescription (identification of medicine) and dosage (in quantity and frequency) were correct.In the absence of a sick child, one with mild symptoms or whom the CHW had recently seen was sought. In the absence of that, a customized simulation to improve insufficiencies in CHW’s performance was employed using a healthy child, recruited with her/his caregiver.Five *Care of Sick Children* forms from the CHW the previous month were randomly selected and reviewed.	First, a data collector observed a CHW examining a sick child who met inclusion criteria (child between 2 and 59 months, symptoms relevant to iCCM, first consultation by CHW for the episode, mother or companion aged at least 18 years and those aged 15 to 17 years who were married or had at least one child) and recorded the CHW’s actions based on the MoH protocol (assessment of symptoms and danger signs, classification, treatment, or referral).Then, the data collector held an exit interview with the child’s companion to ascertain how well they had had comprehended the instructions. After the exit interview, a clinician who had not done the observation conducted a re-exam to avoid bias.In the absence of two sick children spontaneously presenting for care, study teams and CHWs were trained to go to the village and find sick children in the community.The CHW counted the child’s respiratory rate during the exam and the clinician counted it during the re-examination. Counting was considered correct when the CHW’s measurement was within ±5 counts of the clinician’s.Treatment by the CHW was considered correct if both the prescription (identification of medicine and dosage for quantity and frequency) was correct.
**Number of children observed receiving care from a CHW**	
Target of one case per CHW.	Target of two cases per CHW.
**Data collection**Used MoH supervision form to assess the QoC for sick children (classification and treatment).Data collected in paper forms and kept at the PHC to which the CHW is attached. The District Counsellor ensured complete data collection during supervision and entered data later in MS Excel. Data from all districts was compiled by the program’s Monitoring and Evaluation (M&E) Officer of the Malian Red Cross.	Used RADAR data collection forms to assess steps of correct classification and treatment.Observation data collected in paper forms and then entered into an electronic database for analysis; other data collected directly on tablets using ODK Collect Data quality assurance and data cleaning included checking for any doubles with the same site and CHW name, labelling, and categorizing variables, using a reproducible ‘Do’ file in Stata, looking for missing values, or things that were either much too high or much too low, and verifying 100% of paper forms with the data entered.
**Data analysis**Descriptive analyses were done using MS Excel by the M&E Officer.Enabled findings on correct classification and treatment by district, but not by illness.Enabled examining association between correct classification and treatment with CHW sex only.	Enabled findings on correct classification and treatment, by illness, and by district. Enabled examining association between correct classification and treatment and factors such as CHW sex, age, level of education, level of experience, and age of child.

## The RADAR evaluation

The RADAR evaluation, implemented between July and August 2018, was a cross-sectional evaluation carried out on a sample of 300 CHWs that used two rigorous RADAR project tools to evaluate the implementation strength and QoC of the healthcare services that CHWs provided [10]. The implementation strength assessment collected data on CHWs’ sociodemographic, training, and professional characteristics and readiness (including assessing their stock of key supplies and medicines). To assess QoC, the data collector observed as the CHW conducted two sick child consultations. The data collector then held an exit interview with the child’s companion to ascertain how well instructions provided had been comprehended. After the exit interview, a clinician from the team re-examined the child.

## Selection of the supervision cycle to compare with RADAR evaluation

Because each supervision cycle took several months to complete, the start and end dates varied across the six districts. The second and third cycles overlapped with the RADAR evaluation. Cycle 3 data, due to its chronological proximity and higher sample size of CHWs (N = 440), was considered optimal for comparison to the RADAR evaluation data. Figure 1 shows the program’s milestones for supervision cycles in relation to the RADAR evaluation and Supplement Table 2 provides more information on the number of CHWs supervised per district.

## Methodologies of RADAR Evaluation and Supervision to Assess the Quality of Care

Table 1 compares the methodologies of program supervision and the RADAR evaluation and Supplement Table 2 shows additional comparison between both methods. Supplement Figure 2 shows a flowchart of illness diagnosis and treatment in iCCM.

**Table 2.** Program supervision findings on correct assessment, classification, treatment, and dosage of child illnesses.

Indicator	Cycle 3 (%)
**Records review of sick children’s cases from previous month**	
% of sick children seen by CHWs with concordance between signs/symptoms and referral in all five reviewed records (n = 300/440)	68.2
% of sick children seen by CHWs with concordance between signs/symptoms and classification in all five reviewed records (n = 307/440)	69.7
% of sick children seen by CHWs with concordance between the age of child and dosage prescribed for all illnesses in all records reviewed (n = 306/440)	69.5
**Direct observation during case management or simulation**	
% of sick children who had their temperature measured correctly (408/427)	94.4
% of sick children who had their arm circumference measured by CHWs according to MoH protocol (350/424)	82.5
% of sick children examined by CHWs for danger signs, among a list of 14, during consultation (336/432)	77.8
% of sick children who had their respiratory rate counted by CHWs within +/-2 of the clinician’s count (n = 332/375)	88.5
% of sick children who had CHWs use a rapid diagnostic test (RDT) according to MoH protocol (n = 290/385)	75.3
% of sick children who had CHWs correctly classifying their symptoms according to MoH protocol (379/435)	87.1
% of sick children who had CHWs administer the first dose of medicine (n = 325/431)	75.4
% of sick children who had CHWs explain to the mother how to administer the medicine at home (how many tablets/spoonsful, how many times/day and how many days) (n = 332/431)	77.0

**Figure 2.**
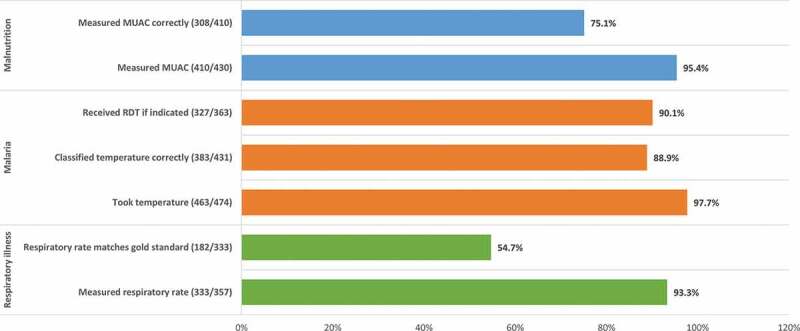
Sick children assessed correctly by CHW for iCCM illness during RADAR evaluation.*Note*: indicated = history of fever as reported by companion, or temperature ≥ 37.5°C, and no RDT within past 15 days.

## Case management by CHWs in 2018

Of the 66,654 sick child consultations carried out by CHWs in 2018, 38,887 (58%) were for uncomplicated malaria, 12,584 (19%) for cough/cold, 7,762 (12%) for pneumonia, and 7,421 (11%) for uncomplicated diarrhea, a pattern observed in subsequent supervisions.

## Results

This section presents findings from the program supervision and RADAR evaluation on the characteristics of CHWs, the program’s implementation strength, the quality of care provided by the CHWs, and the impact of the RADAR evaluation on program supervision.

Supplement Table 4 shows the characteristics of the CHWs assessed in the RADAR evaluation.

## Implementation strength

The RADAR evaluation found that 98.3% of the CHWs received training with sick children and 81.0% received a supervision visit during the previous three months.

Nearly every CHW had pneumonia and uncomplicated malaria medications in their kit. Both the RADAR evaluation and program supervision data found CHWs had the necessary equipment, records/registers, and adequate storage conditions for medications. The RADAR evaluation found medication availability for malaria and pneumonia to be higher than those for diarrhea, cough, and cold, a finding consistent with all supervision cycles since the MoH strategy prioritized commodities for malaria and pneumonia.

## Correct assessment, classification, and treatment of illnesses

**Figure 3.**
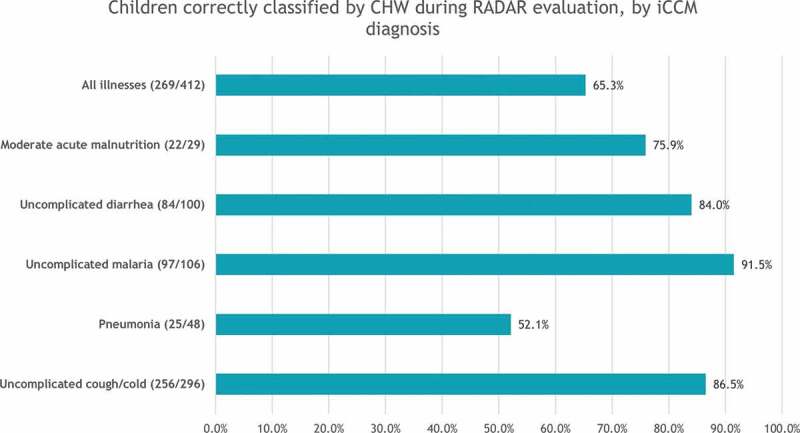
Children correctly classified by CHW during RADAR evaluation, by iCCM diagnosis.

**Figure 4.**
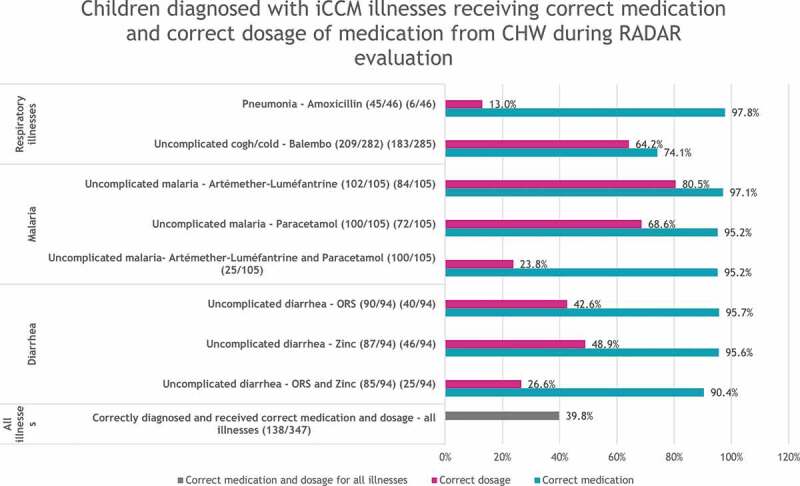
Children diagnosed with iCCM illnesses receiving correct medication and correct dosage of medication from CHW during RADAR evaluation.

A snapshot of key findings on assessment, classification, and treatment by CHWs is presented in Figures 2, 3 and 4 from the RADAR evaluation and in Table 2 from supervision cycle 3. Among the 474 sick children who received care from CHWs in the RADAR evaluation, 65.3% of children were correctly classified across all illnesses, ranging from 91.5% for uncomplicated malaria to 52.1% for pneumonia. Supplement Tables 5, 6 and 7 cite detailed findings.

We found six program supervision indicators that were comparable to indicators from the RADAR evaluation, with three significant differences ranging between 22 and 34 percentage points.

The RADAR evaluation uncovered deficiencies that the program had not been aware of, because the MoH’s data collection form does not capture correct classification and treatment by individual illness type, but only for all illness combined. It also does not distinguish between correct medication and dosage when assessing correct treatment. After the RADAR evaluation findings were shared with them, program staff developed and implemented a plan of action to address these deficiencies, including a refresher course, reinforcement during subsequent supervisions, using monthly PHC meetings to coach CHWs, and a shift from focusing on the composite indicator to assessing individual skill areas.

## The impact of the RADAR evaluation on program supervision

The program staff identified four benefits from the RADAR evaluation for iCCM program supervision.

The first benefit was to initiate customized CHW supervision and coaching. For each supervision session after the RADAR evaluation, the supervision team reviewed the evaluation findings showing areas requiring improvement. They then reviewed the last supervision report of the CHW, which is evaluated to compare scores. If the CHW’s score of the corresponding indicator was low, the supervisor provided coaching on the knowledge and skill area that was lacking, practicing with the CHW repeatedly. The program’s Monitoring and Evaluation Officer supported the process by tracking all supervision reports and informing the supervisors on scores comparable with the RADAR evaluation lower performance areas.

The second benefit was to complement CHW coaching during supervision visits with additional technical support during the monthly meetings at the PHCs when CHWs came to replenish their stock and submit their reports. Historically, these monthly meetings were seen as a platform to reinforce competencies; however, CHWs’ attendance was less consistent among those PHCs and their community health associations that were less capable of organizing them and less financially able to incentivize CHWs to attend, by covering their travel costs and offering refreshments. Therefore, the program offered one-time funding to these PHCs, enabling a more equitable opportunity for all CHWs to improve their knowledge and skills through refresher sessions that target their specific deficiencies.

Although the fund existed only for the first fiscal quarter after the RADAR evaluation, the program leveraged this opportunity to advocate that all PHCs and their community health associations provide CHWs with a per diem to enable consistent attendance at subsequent monthly meetings and many agreed to do this. The program staff felt the RADAR evaluation findings reinforced these institutions’ responsibility to invest systematically in strengthening the competencies of their CHWs.

The third benefit was to strengthen the competency of MoH supervisors. Testing of supervisors at the beginning of the program revealed technical areas that required improvement. The RADAR evaluation showed a correlation between the knowledge and skills gaps of supervisors and that of the CHWs under their responsibility. In response, program staff initiated a joint review of the RADAR evaluation findings with the supervisors and asked them to lead a refresher course for CHWs during the monthly meetings. This created an opportunity for supervisors to review the iCCM subject matter. As a result, program staff observed an improvement in the knowledge and skill level of supervisors and in the overall quality of supervision.

The fourth benefit was to strengthen the relationship between CHWs and their PHC supervisors. While initial training promoted the connection between CHWs and their supervisors, the RADAR evaluation provided an opportunity for the program to reiterate that a CHW’s first point of reference for technical issues was their supervisor. Additionally, the informal contact opportunities that CHWs had with their supervisors beyond routine supervision visits (such as the monthly meetings and phone calls) improved CHW performance. The program tasked the District Counselors, who provided technical support to the MoH staff overseeing iCCM in the field, by reinforcing the relationship between the CHWs and their supervisors, and supported the development of an individual CHW learning pathway that could be monitored consistently.

The quality of supervision and CHW performance improved steadily for the remainder of the program. By Cycle 5, eight indicators on correct classification and treatment for all CHWs improved, on average, to 80% (MoH threshold) or higher.

A socio-demographic survey conducted among all 441 CHWs in 2019 prior to disseminating the findings from the RADAR evaluation found 67.9% of CHWs contacted their supervisors ‘to seek information on technical issues’ (50.9% once a month and 17.0% once a week). In another survey that sampled 195 CHWs in December 2020 as part of the program’s final evaluation, 85.1% of CHWs reported receiving feedback from supervisors during supervision visits and rated the feedback as ‘excellent’ (38.7%) and ‘good’ (55.7%). CHWs also reported that supervisors corrected them when they saw them making a mistake ‘all the time’ (70.8%) and ‘sometimes’ (29.2%).

## Discussion

The independent evaluation gave a detailed picture of the strengths and weaknesses of the QoC CHWs were providing, creating an opportunity to improve both the QoC and program supervision.

## Overall comparison between findings from program supervision and the RADAR evaluation on the quality of care

Both program supervision and the RADAR evaluation measured the QoC provided by CHWs when assessing symptoms and danger signs, classifying illnesses, and treating or referring patients through observation of case management; program supervision carried out an additional assessment by reviewing the records of sick children. The RADAR evaluation provided the findings for each assessment step by illness, and the findings for treatment by the type of medication and dosage. Program supervision provided findings about each assessment for all illnesses combined and, because of the limitations of MoH’s data collection form, also combined the findings about medication and dosage.

The RADAR evaluation showed that CHWs have good skills for correctly classifying uncomplicated malaria, diarrhea, cold, and cough, but not for pneumonia, and that they are skilled in prescribing the correct medication for all illnesses but were weak in prescribing the dosage, except for antimalarial medication. In contrast, program supervision data showed that the competencies of CHWs were acceptable overall, but the results were skewed because CHWs were more skilled at managing malaria, which is predominant for children in Mali (the RADAR evaluation reported that 91.5% of children with uncomplicated malaria were correctly classified and 80.5% received antimalarials). The use of a rapid diagnostic test for malaria must have contributed to the 91.5% rate of correct classification measured by the RADAR evaluation [13].

## Variances in indicator values for quality of care between program supervision and the RADAR evaluation

We found six comparable indicators of QoC between program supervision and the RADAR evaluation (Table 3). There was a difference of three percentage points between both methods for correct measurement of temperature and correct measurement of mid-upper arm circumference. The remaining four indicators were given higher values by program supervision than the RADAR evaluation, with variances between 10 and 34 percentage points (correct counting of respiratory rate 88.5% vs. 54.7%, correct classification of all illnesses 75.4% vs. 65.3%, CHWs administering the first dose 75.4% vs. 65.0%, and correct dosage for all illnesses 69.5% vs. 39.8%, respectively).

**Table 3.** Comparable indicators from RADAR evaluation and program supervision.

Indicator*	RADAR evaluation (%)		Supervision Cycle 3 (%)		Variance
	n	%	n	%	Percentage point
Children who had their temperature measured by the CHW correctly	463	97.7	408	94.4	3.3
Children who had their respiratory rate measured by the CHW correctly	182	54.7	332	88.5	33.8
Children whose mid-upper arm circumference (MUAC) was measured by the CHW according to the protocol	308	86.1	350	82.5	−3.5
Children who had the CHW correctly classify their illness	269	65.3	379	87.1	21.8
Children who had the CHW administering the first dose of all required treatments	407	65.0	325	75.4	10.4
Children who received correct treatment for all illnesses from the CHW	347	39.8	332	69.5	27.7

*Note: The indicators from RADAR evaluation and supervisions have been realigned for comparability.*

Several hypotheses could explain these differences. The timing of data collection and seasonal illness could account for the variance in correct classification and treatment. The RADAR evaluation, which was conducted between July and August 2018, showed that 71.8% of the cases of examined children were for cough and cold, 25.7% were for malaria, 24.3% for diarrhea, and 11.7% for pneumonia. Program data from 2018 showed that CHWs assessed 58.3% for malaria, 19% for cough and cold, 12% for pneumonia, and 11% for diarrhea. Cycle 3 spanned more than 15 months (May 2018 to July 2019), but 95% of CHWs had been supervised by December 2018. The malaria season is from July to October, peaking in August, while the season for cough and cold is from July to January. The RADAR evaluation showed that CHWs were more skilled in managing uncomplicated malaria, so it is possible that most of the cases assessed for QoC by program supervision were for malaria.

The variance in correct respiratory counting could be attributed to the different counting methods used. In program supervision, the CHW and the supervisor counted simultaneously. In the RADAR evaluation, the CHW counted first and the data collector registered the count. After the CHW finished examining the child, an exit interview with the child’s companion was held, followed by the re-examination of the child by a clinician, counting the respiratory rate and comparing it with the CHW’s. The time lag between both counting methods could have been a factor. The differing competencies of supervisors in assessing correct respiratory counting could also have resulted in inaccurate counting. Moreover, CHWs could have felt more relaxed while being observed by their own supervisor than by unknown personnel assessing their competencies in the more formal RADAR evaluation.

## Factors impacting the quality of care provided by CHWs

One year after completing their initial training and after receiving two to three supervisions, some CHWs were still unable to correctly classify and treat children. The RADAR evaluation showed that 52.1% of children were classified correctly for pneumonia and that correct dosage for amoxicillin, oral rehydration salt, and zinc was 13.0%, 42.6%, and 48.9%, respectively. It is not clear why these deficiencies occurred. One hypothesis is that CHWs may not have been optimizing the job aids they were given. Program supervision Cycle 3 showed that 92.8% of CHWs had a copy of the *Care in the Community Guide* and 95.8% had the *Care of Sick Child Form*, both of which included guidance on correct classification and correct prescription of medication type and dosage. Project staff observed that the form was complex, potentially preventing CHWs with lower literacy skills from mastering the content (Supplement Figure 3).

Overall, the RADAR evaluation found that 39.8% of sick children presenting with iCCM illnesses were correctly treated, ranging from 80% for treatment of uncomplicated malaria to 13% for pneumonia. It found that CHWs prescribed correct medications at high rates for all illnesses, ranging from 90.4% for a combination of oral rehydration salt (ORS) and zinc to 97.8% for pneumonia. However, correct dosage was different, ranging from 13.0% for amoxicillin for pneumonia to 82.5% for artemether-lumefantrine for uncomplicated malaria.

The RADAR evaluation found that only 15.9% of sick children cases received correct classification, medication, and dosage. In addition, all MoH training tools, forms, and manuals were in French, which is not the first language of CHWs and could have limited their use.

Several studies have shown various findings on the effectiveness of program interventions to improve the QoC provided by CHWs, particularly the role of supervision. For example, two related studies on the Optimization of Health Extension Program Intervention in Ethiopia – which included training, supportive supervision, and performance reviews of health extension workers, the Ethiopian equivalent of CHWs, (HEWs) – found that the interventions were neither associated with an improved classification of childhood illnesses by HEWs nor improved the use of their services [14,15]. The studies attributed that to complex interventions, delays in implementation, and a short implementation period. An earlier study of Ethiopia’s Health Extension Program showed that performance review and a clinician mentoring meeting, accompanied by follow-up training, increased the odds of correct management of childhood illnesses; however, supervision did not significantly affect the odds of receiving correct care [16].

The Last 10 Km Project in Ethiopia focused on and evaluated the use of job aids by HEWs in their supervision programs. Supervisors asked HEWs to refer to their job aids to find the correct answers to a standard set of questions on classification and treatment of key childhood illnesses [17]. Such program supervision resulted in a significant improvement in the skills of HEWs over time [8].

A systematic review of diverse mentoring approaches in Africa found that mobile mentoring, both within the health facility and remotely by phone, improved competencies in clinical management of childhood illnesses [18].

## Benefits of the external evaluation on improving the quality of care and program supervision

The independent evaluation provided a detailed snapshot of the strengths and weaknesses of the iCCM program in Mali. It guided the development of a plan of action to address the weaknesses through refresher training, program supervision tailored to CHWs’ needs, systematized monthly meetings at health facilities for performance review and remote coaching, and enhanced competence of supervisors in managing childhood illnesses. As a result, the quality of guidance by supervisors continued to improve. By the end of the program, 85.1% of CHWs reported receiving feedback from supervisors during supervision visits and rated the supervisor’s feedback as ‘excellent’ (38.7%) and ‘good’ (55.7%). These CHWs noted that the supervisor corrected them when he or she saw them making a mistake ‘all the time’ (70.8%) and ‘sometimes’ (29.2%).

While the program incorporated several elements of supportive supervision, it provided a limited number of indicators on QoC. A review of the supervision process triggered by the RADAR evaluation indicates that its evaluation of the QoC is agile and credible enough to introduce corrective measures when needed.

## Limitations and further research

The study focused on the quality of care of childhood illnesses implemented through iCCM by CHWs in Mali but did not provide enough detail on the availability of commodities, equipment, and records that were assessed by either the RADAR evaluation or program supervision.

Further research is needed to better understand how initial CHW training can be reorganized to optimize this as a learning opportunity that will enable CHWs to classify and treat illness more quickly, competently, and correctly.

Differences in the assessment methods of the program supervision and RADAR evaluation can make it difficult to compare the estimates for similar indicators. These differences include the use of different tools, processes, and checklists and the amount of focused training for such quality-of-care assessments.

## Conclusion

The study suggests that the closer the estimates are between similar indicators from program supervision data and from a rigorous, external evaluation, the more effective the program supervision in assessing the CHWs’ QoC level and improving it; wider findings indicate that the supervision process requires adjustments. However, methodologies, context, and program interventions need to be factored when interpreting variances.

A rigorous independent evaluation of QoC early in an iCCM program can provide a snapshot of strengths and weaknesses that can be compared with a program’s supervision data. Data from an independent evaluation can be used to assess the effectiveness of program supervision and inform program improvements. Ideally, if resources and time permit, a second independent evaluation at the end of the program to verify whether improvements have been achieved could increase learning on iCCM programs.

The independent evaluation of the Red Cross’ iCCM program in Mali identified strengths and deficiencies. Adjustments to the process enhanced quality of care provided by CHWs and increased the effectiveness of program supervision.

## Supplementary Material

Supplemental Material

Supplemental Material

Supplemental Material

Supplemental Material

Supplemental Material

Supplemental Material

Supplemental Material

Supplemental Material

Supplemental Material

Supplemental Material

Supplemental Material

Supplemental Material

Supplemental Material

Supplemental Material

Supplemental Material

## Paper context

Program supervision is essential to ensure the quality of care of services by community health workers (CHWs) in integrated community case management (iCCM) for childhood illnesses, but its quality is variable. There is limited information on the quality of care in such programs assessed through program supervision. A rigorous assessment of the quality of care in iCCM programs delivered by CHWs could be used as a benchmark to assess the effectiveness of program supervision.

## Data sharing

The data that support the findings of this study are available from the corresponding author [LB] upon reasonable request.
