# Supplementary figures and images for: Comparing program supervision with an external RADAR evaluation of quality of care in integrated community case management for childhood illnesses in Mali
Source: Glob Health Action. 2022 Sep 13;15(Suppl):2006424. doi: 10.1080/16549716.2021.2006424 (PMC9481102; doi:10.1080/16549716.2021.2006424)

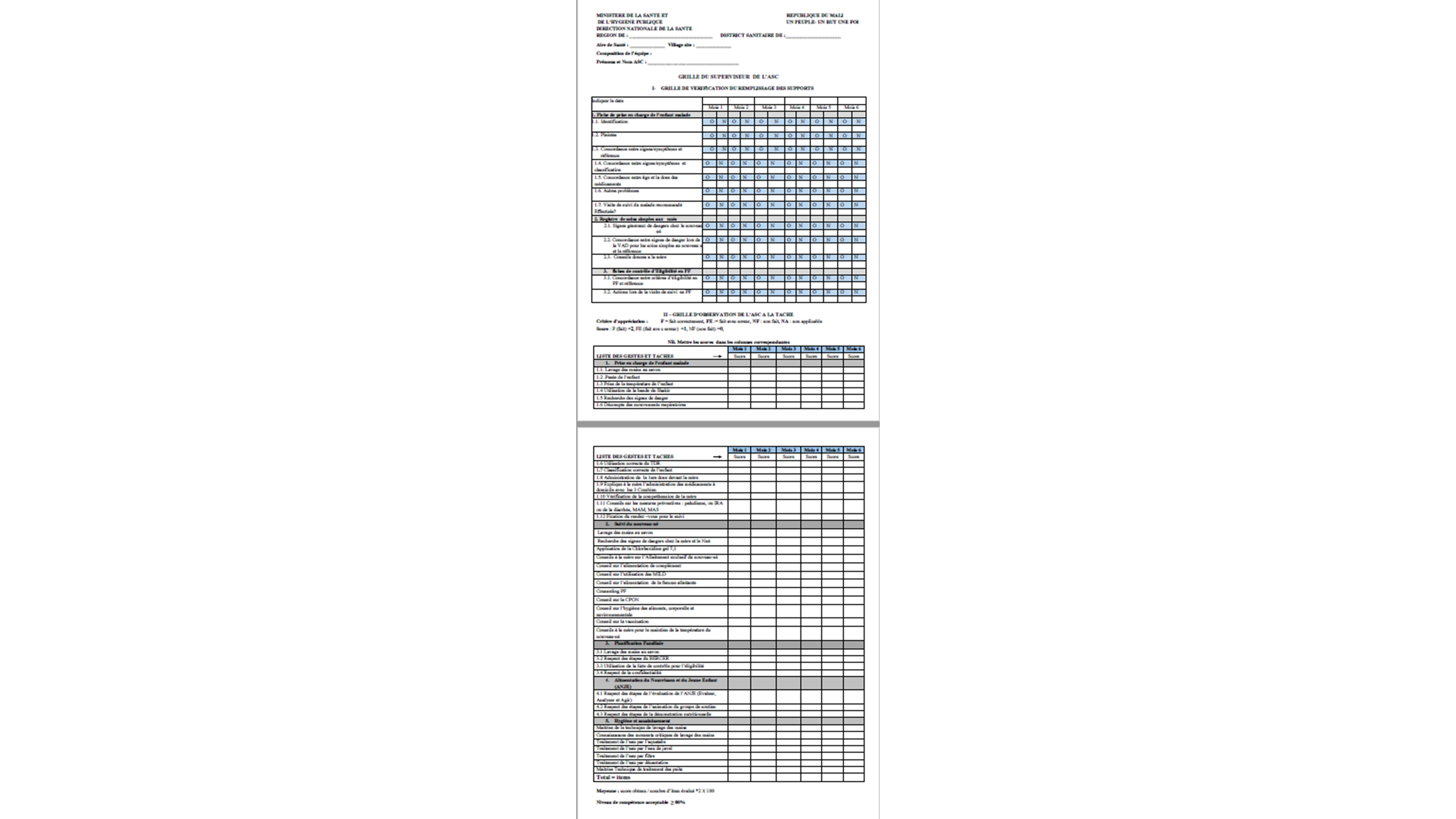

Supplement: Supplemental Material [file ZGHA_A_2006424_SM3949.tif]

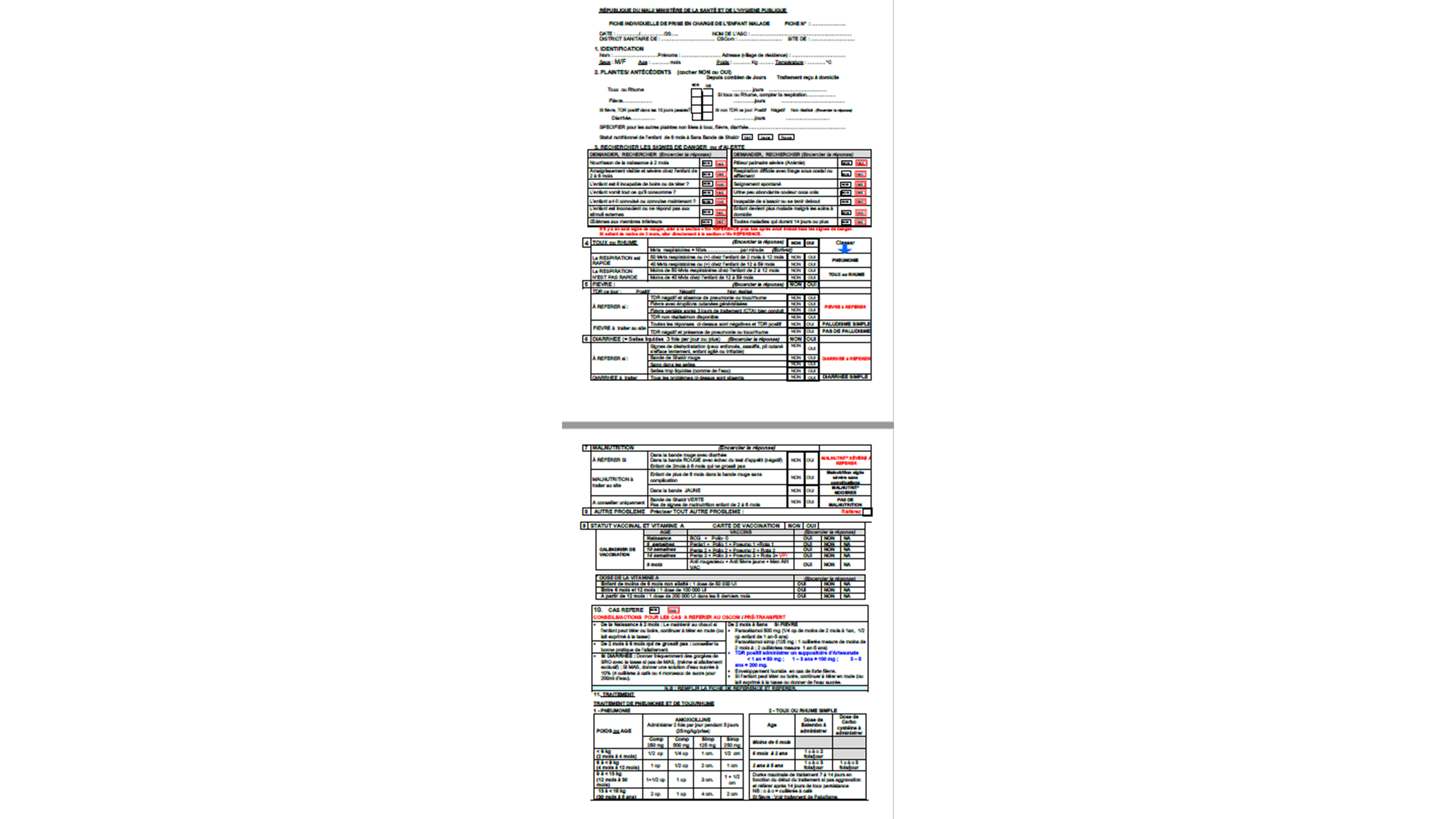

Supplement: Supplemental Material [file ZGHA_A_2006424_SM3838.tif]

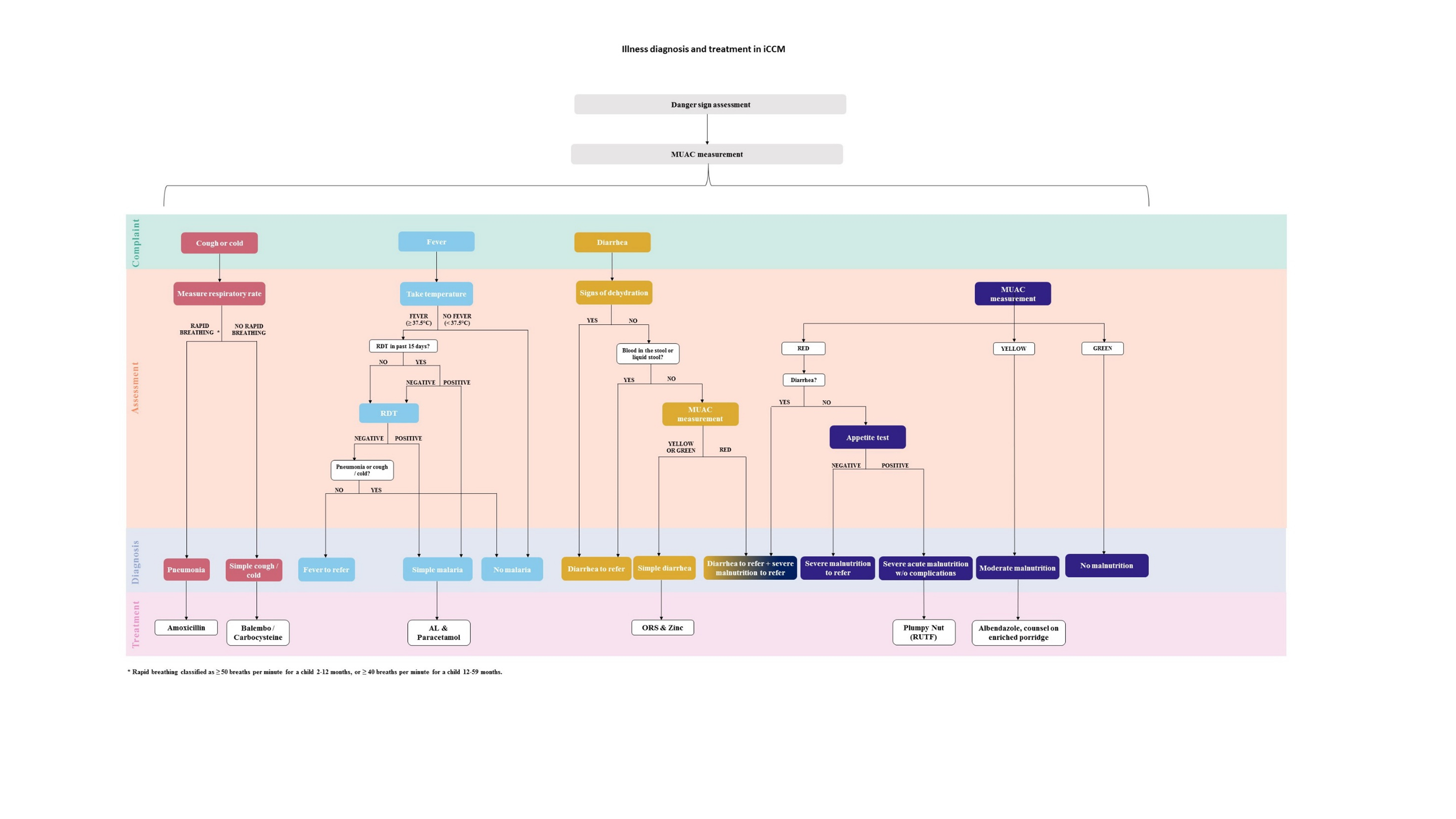

Supplement: Supplemental Material [file ZGHA_A_2006424_SM3821.tif]
