# Supplementary material for: Comparing program supervision with an external RADAR evaluation of quality of care in integrated community case management for childhood illnesses in Mali
Source: Glob Health Action. 2022 Sep 13;15(Suppl):2006424. doi: 10.1080/16549716.2021.2006424 (PMC9481102; doi:10.1080/16549716.2021.2006424)
Supplement: Supplemental Material [file ZGHA_A_2006424_SM3942.docx]

**Supplement Table 7: Results of CHW treatment of sick children during RADAR evaluation**

| Area | Indicator | n/N | Percentage |
| --- | --- | --- | --- |
| Correct treatment, prescription and dosage for respiratory illness | % of children diagnosed with uncomplicated cough/cold by CHW given correct medication (Balembo) by the CHW | 209/282 | 74.1 |
|  | % of children diagnosed with a uncomplicated cough/cold by CHW given correct Balembo and dosage by CHW | 183/285 | 64.2 |
|  | % of children diagnosed with pneumonia who received correct medication (Amoxicillin) by CHW | 45/46 | 97.8 |
|  | % of children diagnosed with pneumonia by CHW who received correct dosage of Amoxicillin by CHW | 6/46 | 13.0 |
|  | % of children not diagnosed with pneumonia by CHW who received Amoxicillin by CHW | 32/370 | 8.6 |
| Correct treatment, prescription and dosage for malaria | % of children diagnosed by the CHW with uncomplicated malaria who received correct medication from the CHW (Artémether-Luméfantrine) | 102/105 | 97.1 |
|  | % of children diagnosed by the CHW with uncomplicated malaria who received correct medication from the CHW (Paracetamol) | 100/105 | 95.2 |
|  | % of children diagnosed by the CHW with uncomplicated malaria who received both Artémether-Luméfantrine and Paracetamol from the CHW | 100/105 | 95.2 |
|  | % of children with negative RDT test result during the consultation who received Artémether-Luméfantrine from the CHW | 5/280 | 1.8 |
|  | Children with uncomplicated malaria who received correct dosage of Artémether-Luméfantrine | 84/105 | 80.0 |
|  | Children with uncomplicated malaria correctly treated with Artémether-Luméfantrine (n=105) |  | 80.0 |
|  | % of children diagnosed with uncomplicated malaria by the CHW who receive correct dosage of Paracetamol by the CHW | 72/105 | 68.6 |
|  | % of children diagnosed with uncomplicated malaria by the CHW given correct Artémether-Luméfantrine and Paracetamol prescription and dosage by the CHW | 25/105 | 23.8 |
|  | Children correctly treated for fever without malaria (n=266) |  | 15.4 |
| Correct treatment, prescription and dosage for diarrhea | % of children diagnosed with uncomplicated diarrhea by the CHW who received correct medication of Oral Rehydration Solution (ORS) from CHW | 90/94 | 95.7 |
|  | % of children diagnosed with uncomplicated diarrhea by the CHW who received correct medication of Zinc | 87/94 | 95.6 |
|  | % of children diagnosed with uncomplicated diarrhea by the CHW who receive both ORS and Zinc from the CHW | 85/94 | 90.4 |
|  | % of children diagnosed with uncomplicated diarrhea by the CHW who received correct dosage of ORS from the CHW | 40/94 | 42.6. |
|  | % of children diagnosed with uncomplicated diarrhea by the CHW who received correct dosage of Zinc from the CHW | 46/94 | 48.9 |
|  | % of children diagnosed with uncomplicated diarrhea by the CHW correctly given ORS and Zinc prescription and dosage by the CHW | 25/94 | 26.6 |
| Correct treatment for all illnesses | % of children correctly diagnosed with any iCCM illness by the CHW who received correct medication and dosage for that/those illness/es from the CHW | 138/347* | 39.8 |
|  | Children who had the CHW administering the first dose of all required treatments (n=407) |  | 65.0 |
| Correct classification and treatment for all illnesses | Correct classification and treatment |  | 15.9 |
|  | Correct classification and incorrect treatment |  | 51.5 |
|  | Incorrect classification and correct treatment |  | 14.9 |
|  | Incorrect classification and treatment |  | 17.6 |

*Fifty-six children were excluded from the analysis because they were referred for danger signs*
