# Supplementary material for: Comparing program supervision with an external RADAR evaluation of quality of care in integrated community case management for childhood illnesses in Mali
Source: Glob Health Action. 2022 Sep 13;15(Suppl):2006424. doi: 10.1080/16549716.2021.2006424 (PMC9481102; doi:10.1080/16549716.2021.2006424)
Supplement: Supplemental Material [file ZGHA_A_2006424_SM3929.docx]

**Supplementary Table 6: Results of CHW classification during RADAR evaluation**

| Area | Indicator | n/N | Percentage |
| --- | --- | --- | --- |
| Respiratory Illness  Correct Classification | % of children classified by the clinician as having a simple cough/cold classified correctly with this condition by the CHW | 256/296 | 86.5 |
|  | % of children classified by the clinician as having pneumonia classified correctly with this condition by the CHW | 25/48 | 52.1 |
| Malaria  Correct Classification | % of children classified by the clinician as having a fever that needs to be referred classified correctly with this condition by the CHW | 2/2 | 100 |
|  | % of children classified by the clinician as having simple malaria classified correctly with this condition by the CHW | 97/106 | 91.5 |
| Diarrhea  Correct Classification | % of children classified by the clinician as having simple diarrhea classified correctly with this condition by the CHW | 84/100 | 84.0 |
| Malnutrition  Correct Classification | % of children classified by the clinician as having moderate malnutrition without complications classified correctly with this condition by the | 22/29 | 75.9 |
|  | % of children classified by the clinician as having severe acute malnutrition without complications classified correctly with this condition by the CHW | 3/4 | 75.0 |
| Correct classification of illness | % of children classified with an iCCM illness whose classification by the CHW matches that of the clinician  * This excludes children referred to the health center for danger signs | 269/412 | 65.3 |

*No children were diagnosed by a CHW with severe diarrhea nor with severe malnutrition.*
