# Supplementary material for: Comparing program supervision with an external RADAR evaluation of quality of care in integrated community case management for childhood illnesses in Mali
Source: Glob Health Action. 2022 Sep 13;15(Suppl):2006424. doi: 10.1080/16549716.2021.2006424 (PMC9481102; doi:10.1080/16549716.2021.2006424)
Supplement: Supplemental Material [file ZGHA_A_2006424_SM3911.docx]

**Supplement Table 5: Results of CHW assessment during RADAR evaluation**

| Area | Indicator | n/N | Percentage |
| --- | --- | --- | --- |
| Respiratory Illness Assessment | % of children presenting with cough or cold whose respiratory rate was measured by CHWs | 333/357 | 93.3 |
|  | % of sick children whose respiratory rate as measured by CHW was within five breaths per minute of the rate measured by study team clinician | 182/333 | 54.7 |
| Malaria Assessment | % of children whose temperature was measured by the CHW | 463/474 | 97.7 |
|  | % of children whose fever classification by the CHW matched that of the clinician | 383/431 | 88.9 |
|  | % of children for whom rapid diagnosis test for malaria (RDT) was indicated* who received RDT from CHW  *indicated = history of fever as reported by companion, or temperature ≥ 37.5°C, and no RDT within past 15 days | 327/363 | 90.1 |
| Malnutrition  Assessment | % of children whose mid-upper arm circumference (MUAC) was measured by CHW | 410/430 | 95.4 |
|  | % of children whose MUAC was measured by the CHW taking all steps according to protocol *  * using the child’s right arm, holding arm at 90° angle to the torso, measuring the band at the mid-point between the shoulder and the elbow, starting the band at 0, and holding the band an appropriate band tightness | 308/410 | 75.1 |
|  | % of children > 6 months not referred to the health center whose MUAC classification by the CHW matched the classification by the clinician | 360/418 | 86.1 |
