# Supplementary material for: Comparing program supervision with an external RADAR evaluation of quality of care in integrated community case management for childhood illnesses in Mali
Source: Glob Health Action. 2022 Sep 13;15(Suppl):2006424. doi: 10.1080/16549716.2021.2006424 (PMC9481102; doi:10.1080/16549716.2021.2006424)
Supplement: Supplemental Material [file ZGHA_A_2006424_SM3893.docx]

| Sociodemographic characteristics | | N | Percentage |
| --- | --- | --- | --- |
| Age (in years) | < 20 | 5 | 2.1 |
|  | 20 – 29 | 131 | 55.3 |
|  | 30 – 39 | 82 | 34.6 |
|  | 40 – 49 | 12 | 5.1 |
|  | 50 and over | 7 | 2.9 |
| Sex | Male | 83 | 35.0 |
|  | Female | 154 | 65.0 |
| Education level | Primary school (1+2) | 103 | 43.5 |
|  | Secondary school | 128 | 54.0 |
|  | Greater than secondary school | 6 | 2.5 |
| Originally from the village | Yes | 11 | 4.6 |
|  | No | 226 | 95.4 |
| Number of years working in the village | < 1 | 39 | 16.4 |
|  | 1 – 4 | 82 | 34.6 |
|  | 5 – 9 | 103 | 43.5 |
|  | ≥ 10 | 13 | 5.5 |
| Experience as a CHW | 2 -11 months | 33 | 13.9 |
|  | 2 years | 36 | 15.2 |
|  | 3 years | 8 | 3.4 |
|  | 4 years | 11 | 4.6 |
|  | 5 years | 27 | 11.4 |
|  | > 5 years | 122 | 51.5 |
