# Supplementary material for: Comparing program supervision with an external RADAR evaluation of quality of care in integrated community case management for childhood illnesses in Mali
Source: Glob Health Action. 2022 Sep 13;15(Suppl):2006424. doi: 10.1080/16549716.2021.2006424 (PMC9481102; doi:10.1080/16549716.2021.2006424)
Supplement: Supplemental Material [file ZGHA_A_2006424_SM3886.docx]

| Program supervision | RADAR Evaluation |
| --- | --- |
| Evaluation of quality of care | |
| - CHWs were evaluated on examining the child for danger signs from a possible list of 14. If at least one danger sign was identified, the CHW stopped the examination and referred the child. - Evaluated correct usage of the rapid diagnostic kit to classify malaria. | - CHWs were evaluated on examining the child for 10 danger signs out of 14, as four were deemed not applicable to the sample. If at least one danger sign was identified, the CHW stopped the examination and referred the child. - Evaluated correct use of the rapid diagnostic kit to classify malaria. |
| Medication | |
| Efforts were made to ensure the availability of medication prior to the supervision visit. In case of a stockout, the CHW referred the child to the PHC. A simulation was conducted by the supervisor to assess correct medication and dosage or another sick child was found whose medication was available at the site. | The team brought with them a complete kit of medication for the CHW to choose from, in accordance with the MoH protocol. |
| Data collection | |
| Database had built-in formulas to identify illogical and missing entries which the M&E Officer followed-up on with District Counsellors.  Data was saved on a password-protected computer used by the M&E | Data collected on paper were stored in a locked cabinet for two years after publication of study results. The database is password protected and is to be stored for two years after publication of study results. |
| Data analysis | |
| - Presented correct classification and treatment findings by cases of sick children, which also equals number of CHWs (example: “% of sick children with correct classification by CHWs” is the same as ”% of CHWs with correct classification of sick children,” since each CHW was supervised for one case). - After each supervision cycle, District Counsellors verified findings with CHWs and analyzed the data with MoH staff and program coordinators, using it to inform implementation. | - The analyses were done in a workshop attended by the stakeholders in January 2019. - Presented the findings of correct classification and treatment by cases of sick children (example: % of sick children with correct classification by CHWs). - The data was analyzed by the stakeholders and initial findings used to inform implementation. |
