# Supplementary material for: Comparing program supervision with an external RADAR evaluation of quality of care in integrated community case management for childhood illnesses in Mali
Source: Glob Health Action. 2022 Sep 13;15(Suppl):2006424. doi: 10.1080/16549716.2021.2006424 (PMC9481102; doi:10.1080/16549716.2021.2006424)
Supplement: Supplemental Material [file ZGHA_A_2006424_SM3880.docx]

**Supplement Table 2: Program supervision of CHWs between October 2017 and September 2020**

| District | # of CHWs in District | Number of CHWs supervised per Cycle | | | | | | |
| --- | --- | --- | --- | --- | --- | --- | --- | --- |
|  |  | 1^st^ | 2^nd^ | 3^rd^ | 4^th^ | 5^th^ | 6^th^ | 7^th^ |
| Banamba | 75 | 75 | 73 | 75 | 75 | 75 | 75 | 75 |
| Dioila | 113 | 113 | 113 | 112 | 111 | 113 | 100 | 113 |
| Kolokani | 68 | 65 | 61 | 68 | 45 | 68 | - | - |
| Koulikoro | 73 | 73 | 68 | 73 | 66 | 72 | 70 | 72 |
| Nara | 25 | 22 | 11 | 25 | 24 | 23 | - | - |
| Sikasso | 87 | 87 | 81 | 87 | 34 | 38 | 87 | 87 |
| All | 441 | 435 | 407 | 440 | 335 | 389 | 332 | 347 |
