# Supplementary material for: Comparing program supervision with an external RADAR evaluation of quality of care in integrated community case management for childhood illnesses in Mali
Source: Glob Health Action. 2022 Sep 13;15(Suppl):2006424. doi: 10.1080/16549716.2021.2006424 (PMC9481102; doi:10.1080/16549716.2021.2006424)
Supplement: Supplemental Material [file ZGHA_A_2006424_SM3873.docx]

**Supplement Table 1: Indicators from supervision relevant to the RADAR evaluation to enable a comparison between both evaluations**

| Area | Supervision Indicator |
| --- | --- |
| Records review | 1. Concordance between signs/symptoms and referral 2. Concordance between signs/symptoms and classification 3. Concordance between age of child and the medication dose |
| Observation of case management | 1. Correct measurement of temperature 2. Correct measurement of arm circumference 3. Search for danger signs 4. Measure respiratory rate 5. Correct utilization of rapid malaria test 6. Correct classification 7. Administration of 1^st^ dose in front of the mother 8. Explain to the mother how to administer the medication at home |
